# Influence of Bilberry Extract on Neuronal Cell Toxicity

**DOI:** 10.3390/biology13060376

**Published:** 2024-05-25

**Authors:** Svenja König, Tamara Bakuradze, Sandy Jesser, Harshitha Ashoka Sreeja, Max J. Carlsson, Jörg Fahrer, Stefan Kins, Elke Richling

**Affiliations:** 1 Division of Human Biology and Neurobiology, Department of Biology, University of Kaiserslautern—Landau, Erwin-Schrödinger-Straße 13, D-67663 Kaiserslautern, Germany; skoenig@rptu.de (S.K.); sreeja@rptu.de (H.A.S.); kins@rptu.de (S.K.); 2 Division of Food Chemistry and Toxicology, Department of Chemistry, University of Kaiserslautern—Landau, Erwin-Schrödinger-Straße 52, D-67663 Kaiserslautern, Germanysandy.jesser@web.de (S.J.); carlsson@chemie.uni-kl.de (M.J.C.); fahrer@chemie.uni-kl.de (J.F.)

**Keywords:** anthocyanins, cell viability, cellular ROS level, amyloid-beta, neuroprotection

## Abstract

**Simple Summary:**

In our study, we used well-established cell models to investigate the influence of a bilberry extract prepared from lowbush blueberries and a fraction of it containing anthocyanins (red pigments with antioxidant properties). Most importantly, our study shows that bilberry extracts containing anthocyanins show a protective antioxidant effect in an Alzheimer’s disease neuronal cell model, underlying the beneficial potential of fruits in diet.

**Abstract:**

Increased intake of dietary antioxidants such as anthocyanins, which are enriched in colourful fruits, is a promising alternative to reduce the risk of degenerative diseases such as Alzheimer’s Disease (AD). Since Amyloid β (Aβ) is one of the key components contributing to AD pathology, probably by reactive oxygen species (ROS) induction, this study investigated the preventive effect of anthocyanin-rich bilberry extract (BE) and its anthocyanin fraction (ACN) on ROS generation and cell toxicity. The results showed a significant and concentration-dependent decrease in neuroblastoma cell (SH-SY5Y) viability by BE or ACN, whereas no cell toxicity was observed in HeLa cells. Incubation with BE and ACN for 24 h diminished the generation of induced ROS levels in SH-SY5Y and HeLa cells. In addition, low concentrations of BE (1–5 µg/mL) showed protective effects against Aβ-induced cytotoxicity in SH-SY5Y cells. In conclusion, our results suggest antioxidant and protective effects of BE and ACN, which could potentially be used to delay the course of neurodegenerative diseases such as AD. Further studies are needed to clarify the high potential of anthocyanins and their in vivo metabolites on neuronal function.

## 1. Introduction

A diet rich in fruits and vegetables has long been associated with numerous health benefits, and one of the key components responsible for these effects are anthocyanins [1,2]. Anthocyanins are water-soluble secondary metabolites found naturally in many fruits, particularly those with deep red, purple, or blue colours [3]. Consumption of fruit juices rich in anthocyanins from wild blueberries (bilberries), which are among the most abundant dietary sources of anthocyanins [4,5,6], has been shown to positively impact lipid metabolism, DNA protection, and exhibit anti-inflammatory and antioxidative properties [7,8,9,10]. This suggests that anthocyanins have beneficial effects in various diseases like cardiovascular diseases (CVD), diabetes, cancer, and neurodegeneration [11,12,13]. As the risk of Alzheimer’s Disease (AD) is also associated with risk factors of CVD and metabolism diseases, anthocyanin intake is assumed to decrease the risk of dementia disorders in late age [12,14,15]. Indeed, several lines of evidence suggest that anthocyanins and anthocyanin-rich food can modulate brain functionalities and age-related neuronal degeneration [16,17], influencing inflammation, oxidative stress, excitotoxicity, and altered neurotransmission [16]. Blueberries are especially highlighted to improve cognitive functions such as memory in humans [18,19,20] or rats associated with a reduction of oxidative stress and excitotoxicity [21]. Although the exact cause of neurodegeneration in Alzheimer’s disease remains unclear, it is well known that accumulation of amyloid-β (Aβ) leads to the development of plaques [22,23]. Moreover, Aβ may trigger an increase in the production of reactive oxygen species (ROS) in neuronal cells that are particularly sensitive to ROS, thereby promoting cell death [24,25]. 

Based on these observations, the present study investigates the effects of anthocyanin-enriched bilberry extracts on ROS formation and cytotoxicity in neuronal cells (human neuroblastoma cell line; SH-SY5Y) and non-neuronal epithelial cells (HeLa). Furthermore, we studied cell viability and putative cell death induction (apoptosis and necrosis) in cells treated with low extract concentrations. In addition, the potential beneficial effects of the bilberry extract in protecting against oxidative stress, associated with increased levels of Aβ and further causing cell death, were investigated.

## 2. Material and Methods

### 2.1. Materials

All chemicals and reagents used were of analytical grade. 2′,7′-Dichlorofluorescein diacetate (DCFH-DA), poly-L-lysine, catalase, resazurin sodium salt, and sodium dodecyl sulfate (SDS) were purchased from Sigma-Aldrich (Taufkirchen, Germany). H_2_O_2_ was from VWR International GmbH (Darmstadt, Germany). Aβ_25–35_ was obtained from Thermo Fisher Scientific (Waltham, MA, USA).

SH-SY5Y (ATCC^®^ CRL-2266™) and HeLa (ATCC^®^ CCL-2) cells were obtained from ATCC (Manassas, VA, USA). Dulbecco’s modified essential medium (DMEM), Minimum Essential Medium (MEM), Nutrient Mix F12, MEM non-essential amino acids (NEAA), L-glutamine, fetal calf serum (FCS), penicillin/streptomycin, dimethyl sulfoxide (DMSO), and trypsin were from Thermo Fisher Scientific (Darmstadt, Germany). Cell culture materials were purchased from Greiner Bio-One (Essen, Germany) and VWR International GmbH (Darmstadt, Germany).

### 2.2. Bilberry Extract and Its Anthocyanin Fraction

Bilberry (*Vaccinium myrtillus* L.) extract (BE) was generated from pure fruit juice using Amberlite XAD7 adsorber resin (Sigma Aldrich, Taufkirchen, Germany), and the anthocyanin fraction (ACN) was prepared using Sartobind S IEX 150 mL cellulose membrane (Sartorius, Göttingen, Germany) as described in [26]. Both BE and ACN were provided and characterised by Prof. Dr. Peter Winterhalter (Institute of Food Chemistry, Technische Universität Braunschweig, Braunschweig, Germany). 

### 2.3. Cell Culture 

All cells were cultivated at 37 °C in a humidified atmosphere with 5% CO_2_ and the appropriate cell growth media. Human neuroblastoma cells (SH-SY5Y) were grown in 175 cm^2^ flasks using MEM/Nutrient Mix F12 (1:1) media, supplemented with 10% FCS, 1% L-glutamine, 1% NEAA, 100 U/mL penicillin, and 100 µg/mL streptomycin. Human cervical cancer cells (HeLa) were cultivated in 10 cm culture dishes with DMEM media, supplemented with 10% FCS and 1% penicillin/streptomycin.

For performing the biological tests, the cells were incubated with BE and ACN extracts (1–100 µg/mL dissolved in DMSO, final solvent concentration ≤ 1.0%) in FCS reduced medium (5% FCS) for 24 h. To avoid the generation of extracellular hydrogen peroxide (H_2_O_2_) by pro-oxidative interaction of phenolic compounds with cell-culture media constituents, incubations of cells were performed in the presence of catalase (100 U/mL). 

### 2.4. Cell Viability 

Cell viability was determined by a resazurin reduction assay according to O’Brien et al., 2000 [27], which measures metabolic activity from the reduction of resazurin to resorufin at 530/590 nm. Cells (SH-SY5Y cells: 1.8 × 10^5^ cells/well; HeLa cells: 3 × 10^4^ cells/well) were seeded into PLL-coated clear 48-well plates, cultivated for 24 h, followed by treating the cells for a further 24 h by a substance of interest. Therefore, different concentrations of BE and ACN extracts (1–100 µg/mL dissolved in DMSO, final concentration 1.0%) were diluted into the appropriate cell growth media and applied. In another experiment, SH-SY5Y cells were pretreated with different concentrations of BE (1–5 µg/mL) for 2 h, followed by 24 h exposure to 10 µM Aβ_25–35_. SDS (0.1%) was used as a positive control.

After treatment, the cell media was substituted by serum-free medium (500 µL) containing 10% resazurin solution for 1 h. Fluorescence was measured by a Synergy 2 microplate reader (BioTek Instruments GmbH, Bad Friedrichshall, Germany; ex/em: 530/590 nm, 37 °C). Results were expressed as relative cell viability in percentage of the solvent control.

### 2.5. Cellular ROS Level (Dichlorofluorescein (DCF) Assay)

Intracellular ROS levels were determined in SH-SY5Y and HeLa cells by the DCF assay, according to O’Brien et al. and Fuchs et al., with slight modifications [27,28]. Cells were seeded into PLL-coated black, clear-bottom 96-well plates (SH-SY5Y cells: 2 × 10^4^ cells/well; HeLa cells: 8.5 × 10^3^ cells/well), cultivated for 24 h, and incubated with BE or ACN extracts (1–100 µg/mL dissolved in DMSO, final solvent concentration ≤ 1.0%) in FCS reduced medium (5% FCS) for further 24 h. After removing the medium, cells were incubated for 30 min with 50 mM 2′,7′-dichlorodihydrofluorescein diacetate (DCFH-DA; dissolved in DMSO: 0.5% *v*/*v* in PBS, pH 7.0), washed and incubated with 250 µM TBH or 300 µM H_2_O_2_ in PBS for 30 min. The increase of fluorescence (FI) resulting from oxidation of the non-fluorescent product dichlorofluorescein to DCF by intracellular ROS was measured at 0 and 30 min in a Synergy 2 microplate reader (BioTek Instruments GmbH, Bad Friedrichshall, Germany; ex/em: 485/528nm, 37 °C). FI was calculated as (F_30min_ − F_0min_)/F_0min_ × 100, and results are expressed as relative FI in percentage of TBH or H_2_O_2_ control, respectively.

### 2.6. Annexin V/PI Staining and Flow Cytometry

Detection of apoptosis was measured using Annexin V/PI staining and flow-cytometric analysis as reported [29]. Briefly, SH-SY5Y cells (1 × 10^6^ cells/mL) were seeded into PLL-coated petri dishes, cultivated for 24 h, and incubated with BE or ACN extracts (5–10 µg/mL dissolved in DMSO, final solvent concentration ≤ 1.0%) in FCS reduced medium (5% FCS) for further 24 h. After removing the medium, the cells were washed with PBS and isolated by trypsin (0.5% *w*/*v*). After centrifugation (4 min, 1100 rpm, RT), cell pellets were resuspended in binding buffer (10 mM HEPES/140 mM NaCl/2.5 mM CaCl_2_/0.1% BSA; pH 7.4) containing AnnexinV-FITC (Miltenyi Biotec, Bergisch Gladbach, Germany). After incubation on ice for 15 min, propidium iodide (PI) in binding buffer (10 µL PI and 430 µL binding buffer) was added, and cells were analysed by flow cytometry using a FACS Canto II (BD Biosciences, Heidelberg, Germany) and the corresponding BD FACS Diva software 6.0 (BD Biosciences, Heidelberg, Germany).

### 2.7. Statistics

Results are presented as means and standard deviations (SD) of three to five independent experiments. Origin 2018 (OriginLab, Northampton, MA, USA) was used for statistical analyses. Data from samples treated with BE and ACN were analysed for significant differences (*p* < 0.05, *p* < 0.01, and *p* < 0.001) from either the oxidant-treated control (DCF assay) or the respective solvent control (resazurin assay, apoptosis assay) by unpaired one-sample (one-sided) *t*-test.

## 3. Results

In our study, we used a well-characterised bilberry extract [26], which has a high polyphenol content (58.3 g GAE/100 g), containing 14 different anthocyanin glycosides (AC), including glycosides of delphinidin, cyanidin, petunidin, peonidin, and malvidin [26]. Additionally, the bilberry extract also contained colourless phenolic compounds, commonly referred to as copigments, such as chlorogenic acid, coumaroyliridoid, and quercetin derivatives [26]. The ratio of anthocyanins to copigments in bilberry extracts was found to be equal (50/50%). 

### 3.1. Effects of BE and ACN on Cell Viability 

**Figure 1:**
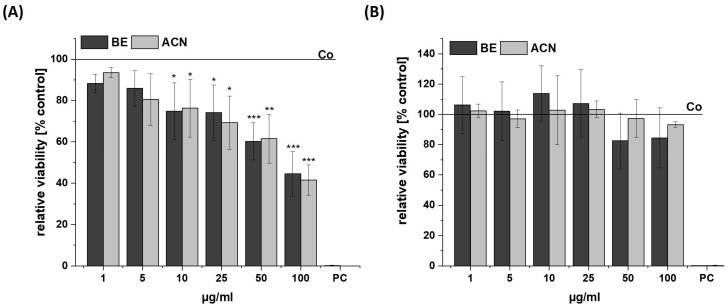
Cell viability in SH-SY5Y (**A**) and HeLa cells (**B**) after 24 h incubation with bilberry extract (BE) or its isolated anthocyanin fraction (ACN) determined by a resazurin assay using concentrations of 1–100 µg/mL. A positive control (PC) was performed using 0.1% SDS. Results are shown as the percent of the solvent control (Co; 1% DMSO), while the Co value is indicated with a horizontal line. Error bars indicate the standard deviation. Significance was calculated against the solvent control using student’s *t*-test: * *p* < 0.05, ** *p* < 0.01, *** *p* < 0.001; *n* = 2–4.

The influence of BE and its ACN fraction on the viability of SH-SY5Y and HeLa cells was determined to ensure that they were used in a non-cytotoxic concentration range (cell viability ≥ 80%) (Figure 1). The cells were incubated for 24 h with BE or ACN using concentrations of 1 µg/mL, 5 µg/mL, 10 µg/mL, 25 µg/mL, 50 µg/mL, and 100 µg/mL prior to resazurin assay. A significant and concentration-dependent decrease of SH-SY5Y cell viability was shown after incubation with both BE and ACN, compared to the solvent control. At low concentrations of both extracts (1–10 µg/mL) tested, the viability of the SH-SY5Y cells decreased from 94 (1 µg/mL) to 75% (10 µg/mL). However, a distinct cytotoxic effect was observed at the highest concentration (100 µg/mL), resulting in a loss of cell viability by 55%. In contrast, BE and ACN caused no significant modulation of cell viability in HeLa cells, even at the highest concentration of 100 μg/mL. This indicates that the extract and its fraction did not influence viability in HeLa cells but at higher concentrations in neuronal SH-SY5Y cells.

### 3.2. Modulation of Cellular Redox Status

**Figure 2:**
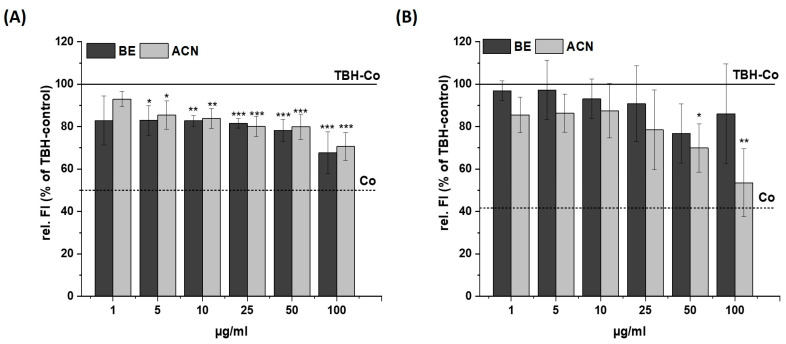
Effects of bilberry extract (BE) and its anthocyanin fraction (ACN) on tert-butyl hydroperoxide (TBH)-induced ROS level in SH-SY5Y (**A**) and HeLa cells (**B**) after 24 h incubation with different concentrations of the extracts (1–100 µg/mL). The relative fluorescence (rel. FI) measured within a DCF-Assay is shown as a percentage of a TBH control (TBH-Co) while the TBH-Co value is shown as a horizontal line and the solvent control (Co; 1% DMSO) as a dashed line. Error bars indicate the standard deviation. Significance to the TBH-Co was calculated using student’s *t*-test: * *p* < 0.05, ** *p* < 0.01, *** *p* < 0.001; *n* = 3–5.

**Figure 3:**
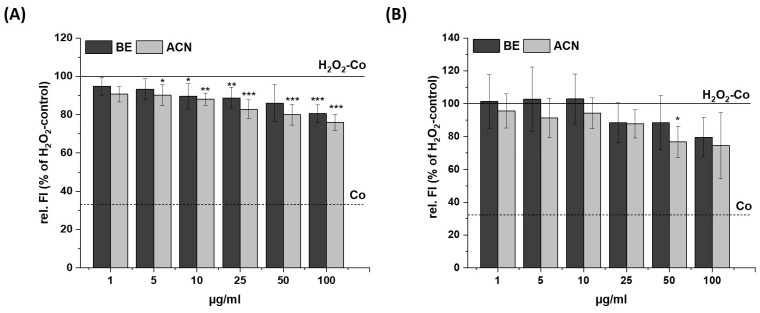
Effects of bilberry extract (BE) and its anthocyanin fraction (ACN) on H_2_O_2_-induced ROS level in SH-SY5Y (**A**) and HeLa cells (**B**) after 24 h of incubation with different concentrations of the extracts (1–100 µg/mL). The relative fluorescence (rel. FI) measured within a DCF-Assay is shown as the percentage of an H_2_O_2_ control (H_2_O_2_-Co), while the H_2_O_2_-Co value is shown as a horizontal line and the solvent control (Co; 1% DMSO) as a dashed line. Error bars indicate the standard deviation. Significance to the H_2_O_2_-Co was calculated using student’s *t*-test: * *p* < 0.05, ** *p* < 0.01, *** *p* < 0.001; *n* = 3–5.

We investigated the preventive effects of BE and ACN on TBH- and H_2_O_2_-induced cellular ROS levels in SH-SY5Y and HeLa cells to test for a possible cell-specific response of neuronal and non-neuronal cells (Figure 2 and Figure 3). 

After incubating SH-SY5Y cells with BE or ACN for 24 h, the TBH-induced ROS levels were significantly reduced to 80–70% in a concentration-dependent manner compared to the positive control (Figure 2A). HeLa cells only showed a trend but no significant reduction in ROS levels when incubated with BE (Figure 2B). However, HeLa cells treated with ACN also showed a significant preventive effect against TBH-induced ROS production at concentrations of 50 µg/mL and 100 µg/mL. The modulation of H_2_O_2_-induced ROS levels in the same cells after 24 h incubation with BE or its ACN fraction (Figure 3) showed comparable results to those of TBH-induced ROS levels, even though the preventive effect was less pronounced. Again, a significant and concentration-dependent reduction of ROS levels was observed in SH-SY5Y cells (Figure 3A). Similarly, ACN was more effective than BE in HeLa cells (Figure 3B). 

Taken together, BE and its anthocyanin fraction showed preventive ROS-reducing effects in human neuroblastoma cells (SH-SY5Y) already at low concentrations, while only ACN at higher concentrations significantly reduced ROS generation in HeLa cells.

### 3.3. Effects of BE and ACN on Cell Death

**Figure 4:**
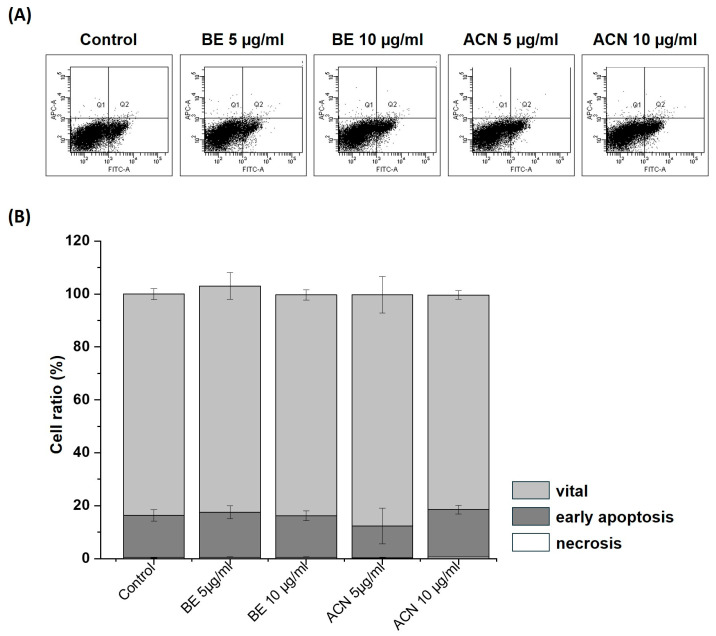
Effects of bilberry extract (BE) and its anthocyanin fraction (ACN) on cell death after 24 h of incubation determined by Annexin V-FITC/PI in SH-SY5Y cells using different concentrations of the extracts (5–10 µg/mL). (**A**) representative flow cytometry diagram of staining with Annexin V-FITC/PI. The vital, early apoptotic, and late apoptotic/necrotic cells were present in the lower left, lower right, and upper right squares, respectively. As a control, the solvent DMSO (0.1%) was used. (**B**) Quantification of the percentage of vital, early apoptotic, and late apoptotic/necrotic cells according to BE and ACN treatment. Error bars represent the standard deviation. *n* = 3.

To exclude the possibility that the observed decrease in cellular ROS levels in SH-SY5Y cells at the lower extract concentrations (5 µg/mL and 10 µg/mL) was due to their protective effect and not due to a reduced number of viable cells, we investigated the influence of BE and ACN on apoptosis and necrosis in SH-SY5Y cells using flow cytometry (Figure 4). The representative flow cytometry diagram of Annexin V-FITC/PI staining of the cells (Figure 4A) and its quantification (Figure 4B) showing the percentage of vital, early apoptotic, and late apoptotic/necrotic cells according to BE and ACN treatment revealed no changes compared to the solvent control. Together, our findings show that low concentrations of BE and ACN do not induce apoptotic or necrotic events, indicating that the observed ROS-reducing effects in SH-SY5Y cells are due to a preventive mechanism of ACN.

### 3.4. Effects of BE and ACN on Aβ_25–35_ Induced Cytotoxicity

**Figure 5:**
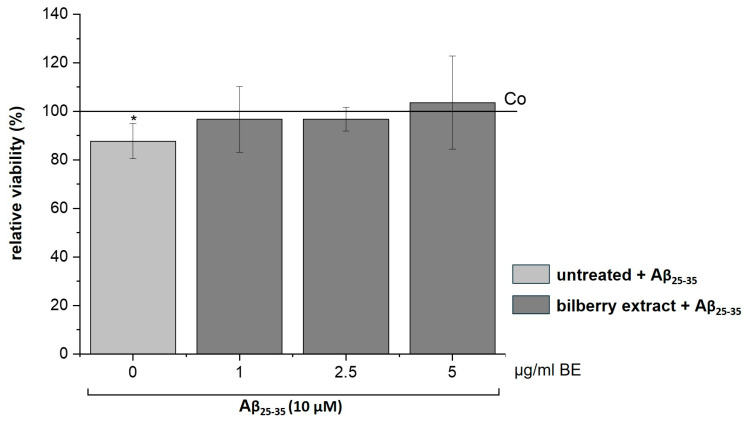
Effects of bilberry extract (BE) on the viability of Aβ_25–35_-treated SH-SY5Y. Cells were pretreated with BE (1–5 µg/mL) for 2 h, followed by 24 h treatment with 10µM Aβ_25–35_. The results of the resazurin assay are shown as the percent of solvent control (Co: 1% DMSO; shown as a horizontal line), indicating a loss of viability via Aβ, which was diminished by BE treatment with different concentrations (1–5 µg/mL). Error bars indicate the standard deviation. Significance according to the solvent control was determined using student’s *t*-test: * *p* < 0.05. *n* = 2–3.

It has been reported that Aβ increases the generation of ROS [30,31], which leads to apoptotic neuronal cell death that can be inhibited by antioxidants [31,32]. Therefore, we assumed a preventive effect of our BE on Aβ-induced cytotoxicity. To test this assumption, we treated SH-SY5Y cells with non-cytotoxic concentrations of BE (1–5 µg/mL) for 2 h, followed by Aβ_25–35_ (10 µM) incubation for 24 h. Interestingly, we observed that BE relieved the Aβ_25–35_-induced loss of cell viability (Figure 5), suggesting a preventive effect of BE on Aβ-induced cell toxicity. 

## 4. Discussion

Oxidative cellular damage, characterised by an imbalance between the production of ROS and antioxidant defences, has been implicated in the pathogenesis of several degenerative diseases, such as CVD and certain types of cancer. ROS and other oxidants have also been described as detrimental factors in neuronal dysfunction and the development of neurodegenerative diseases such as AD [16]. Numerous in vitro and in vivo studies have suggested that consumption of food rich in anthocyanins may help to reduce the risk of the above-mentioned diseases due to the multiple biological effects of anthocyanins, such as antioxidative, anti-inflammatory, anti-atherosclerotic, and anti-carcinogenic effects [12]. 

As the content of anthocyanins is relatively high in bilberries, we investigated in this study the influence of bilberry extract and its anthocyanin fraction on cell viability and cellular ROS levels, using human neuroblastoma SH-SY5Y cells, as well as non-neuronal HeLa cells, in order to identify a cell-specific response between different cell types. A previous study showed that higher concentrations of BE induced apoptosis in HeLa cells; therefore, we used lower concentrations that were reported to be non-cytotoxic [33]. Accordingly, BE, as well as ACN exhibited no cytotoxic effects against HeLa cells. In contrast, BE and the ACN enriched fraction exhibited a dose-dependent cytotoxic effect in neuroblastoma cells, indicating a higher vulnerability of neuronal cells towards the anthocyanin fraction. Interestingly, other studies using blueberry extracts with lower anthocyanin content in even higher concentrations reported no changes in SH-SY5Y cell viability [34], whereas anthocyanins extracted from chokeberry (*Aronia melanocarpa*) even increased cell viability [35]. These data suggest that ACN fractions prepared from different fruit sources using different extraction protocols may differ in their cytotoxicity to SH-SY5Y cells. Unfortunately, the extracts used in the other studies were not analysed in the same detail as our BE extract, thus preventing a more systematic analysis of the potential cytotoxic components in the BE extracts. 

Within our study, investigations on the antioxidative influence of BE and ACN on cellular ROS showed that both revealed a preventive effect against H_2_O_2_ and TBH induced-ROS production in SH-SY5Y cells similar to the application of low amounts of de-alcoholized red wine powder [36], dietary polyphenolic metabolites [37] or individual anthocyanins [38]. In our study, BE showed a less pronounced effect on HeLa cells compared to SH-SY5Y cells, which is in accordance with other studies on cell lines such as HT-29 and Caco-2 colon carcinoma cells that needed even higher concentrations of BE to show a preventive effect against TBH-induced ROS [39]. Taken together, our data suggest that neuronal cells benefit more from the antioxidant effect of ACN than other cell types. Although SH-SY5Y cells are a valuable model to test the neurotoxic properties of bioactive compounds, especially with regard to cell viability, mitochondrial function, and oxidative stress [40], it should be noted that it is a neuroblastoma cell line. As anthocyanins are known to be poorly bioavailable and the activity of the different metabolites found in plasma or tissues such as the brain are rarely analysed [41], future studies are very important. These should either examine individual metabolites in primary neuronal cultures or test the anthocyanin-enriched extracts in vivo in animal models or even in humans (proof-of-concept).

The ROS-mediated cytotoxic effect of Aβ is well known and has already been reported in several in vitro studies [24,25,42,43]. We evaluated the preventive effect of low concentrations (5–10 µg/mL) of BE on Aβ_25–35_ induced cytotoxicity in SH-SY5Y cells and observed an increase in cell survival when cells were pretreated with BE before Aβ application. This indicates that BE counteracts the cytotoxic effect of Aβ, probably by preventing Aβ-induced ROS formation. Likewise, extracts from *Salvia miltiorrhiza* (red sage), *Aronia melanocarpa* (chokeberry), *Centella asiatica*, and other herbal plants protect against Aβ induced cytotoxicity in SH-SY5Y cells [35,44,45,46]. Thus, certain foods, rich in antioxidants, may potentially benefit individuals with Alzheimer’s disease by supporting brain health and reducing inflammation. However, more studies are needed to establish a definitive link between food and Alzheimer’s prevention or treatment [16,17].

In conclusion, our results have demonstrated antioxidant and protective effects of BE and especially ACN fractions in a cell-specific neuronal/non-neuronal manner. Together, our data underline the need for further research to clarify the high potential of ACNs and their in vivo metabolites on neuronal function in the brain. 

## 5. Conclusions

This research revealed cell-type-specific differences in the strength of effects caused by anthocyanin-rich bilberry extract. Thereby, neuronal cells benefit more from the antioxidant effect of the fruit extract, while effects on non-neuronal cells are less pronounced using the same concentrations. Nevertheless, low concentrations of anthocyanin-rich bilberry extract reduced the formation of ROS and further prevented Aβ-induced cell toxicity, probably due to its antioxidant effect. Thus, anthocyanin enriched extracts, especially from bilberries, have a huge potential in slowing down the progression of degenerative diseases such as AD. However, further in vivo studies are needed to assess the future prospects of bilberry-derived antioxidants.

## Data Availability

The data presented in this study are available in the article and on demand.
